# Effect of humic acid supplementation on lamb gastrointestinal health and performance

**DOI:** 10.1007/s11250-026-04882-5

**Published:** 2026-02-12

**Authors:** Diógenes Adriano Duarte Santana, Francieli Rolinski, Leticia Graziela Trombetta, Rafaela Maria Debastiani Göhringer, Tharini Xavier Accioly Kaled, Caroline Ramos dos Santos, Saulo Henrique Weber, Cristina Santos Sotomaior, Rüdiger Daniel Ollhoff

**Affiliations:** 1 Graduate Program in Animal Science, Pontifícia Universidade Católica do Paraná, Curitiba, Brazil; 2 Veterinary Medicine, Pontifícia Universidade Católica do Paraná, Curitiba, Brazil

**Keywords:** Feed additive, Humic substances, Immune cells, Diarrhea, *Haemonchus contortus*

## Abstract

Gastrointestinal infections significantly impact sheep health and productivity, with diarrhea and parasites particularly threatening lambs. To address these challenges, feed additives offer solutions to enhance animal health and performance. This study aimed to investigate the effects of humic acids (HA) on the gastrointestinal tract and growth of lambs. Forty 1/2 Hampshire Down weaned lambs (aged 100.9 ± 7.1 days; average weight 24.4 ± 3.9 kg) were randomly allocated to two groups (20 each), balanced for weight and sex. The control group (CG) received a basal diet without HA supplementation, while the treatment group (TG) was fed a basal diet plus commercial HA at 500 mg/kg body weight/day for 56 days. Every two weeks, lambs underwent hematological, biochemical, parasitological, immunological, fecal consistency, and performance analyses. After the experimental period, 10 lambs per group were slaughtered. Histological analyses included measuring abomasal mucosa thickness and mucus production in the abomasum, duodenum, jejunum, and ileum. Tissues from the abomasum, duodenum, jejunum, ileum, and cecum were also used for leukocyte counts and scanning electron microscopy (SEM) evaluations, while parasitological analysis consisted of parasite recovery from the abomasum and small intestine. The TG exhibited improved fecal consistency, a decrease in CD8 lymphocytes in the abomasum and duodenum, and reduced platelet counts. In general, hematological, biochemical, parasitological, histological, and performance parameters did not differ significantly between groups. HA fragments were not able to be distinguished with SEM on gastrointestinal mucosa surface. Overall, HA supplementation positively impacted digestive health by mitigating gastrointestinal inflammation.

**Supplementary Information:**

The online version contains supplementary material available at 10.1007/s11250-026-04882-5.

## Introduction

Rising consumer demand for sheep meat, dairy products and wool (FAO 2021) is challenged by gastrointestinal infections, which represent a significant threat to the health and productivity of sheep. Gastrointestinal infections result in a reduction in production due to spoilage and mortality (Mariano et al. 2018; Chagas et al. 2022). The control of gastrointestinal infections in small ruminants is dependent on the utilization of antiparasitic or antibiotics. However, there is a mounting concern regarding resistant organisms, which renders the pursuit of efficacious treatments challenging (Martínez-Valladares et al. 2015; Lambertz et al. 2019; Herawati et al. 2023).

Given that gastrointestinal parasitism is a primary challenge in pasture-based sheep production, evaluating potential solutions like feed additives under conditions that mimic this natural challenge is crucial (Chagas et al. 2022). Within this context, feed additives are being actively studied to either reduce the reliance on these drugs or to combine them strategically, aiming to achieve a synergistic effect that enhances both animal health and productivity (Nolinda et al. 2024).

Humic acids (HA) are derived from humic substances, which arise from the natural decomposition of plant (peat) and animal material by soil microbial activity, which results in the formation of complex macromolecules characterized by a high density of acidic functional groups, primarily carboxylic and phenolic groups. They are found in nature as natural deposits of leonardite (oxidized lignite) (Ricca et al. 1993; Stevenson 1994).

HA has been demonstrated to stimulate the immune system and enhance the host’s defence mechanisms against certain microorganisms and their inducers (Jooné and Van Rensburg 2004; Wang et al. 2008; Cetin et al. 2011; Mudroňová et al. 2020). HA has also anti-inflammatory and antimicrobial properties, which are exerted by inhibiting inflammatory pathways, reducing oxidative stress, disrupting pathogen structure, and decreasing virulence gene expression. These combined mechanisms facilitate the regeneration of damaged gastrointestinal mucosa, thereby reducing inflammation, combating harmful bacteria, and supporting a healthier gut environment (EMEA 1999; Islam et al. 2005; Wang et al. 2020a, 2022a, 2023; He et al. 2023).

Several studies have demonstrated that HA supplementation can enhance feed conversion and promote greater weight gain in different domestic species, including pigs (Zralý and Písaříková 2010; Wang et al. 2020a), broiler chickens (Ozturk et al. 2012; Nagaraju et al. 2014) and cattle (Islam et al. 2005; Cusack 2008; Wang et al. 2022b). In small ruminants, the use of HA has also been explored. Studies with goats, for instance, suggested a reduction in parasite fecal egg counts and improvements in ruminal fermentation, although results on productive performance are variable (El-Zaiat et al. 2018; Ikyume et al. 2022). However, the literature on the effects of HA in sheep is still scarce, especially regarding their local mechanisms of action in the GIT (Wang et al. 2020b). Despite the fact that HA do not penetrate animal tissues (Büsing et al. 2014), the question of their absorption remains a subject of controversy (Buesing et al. 2011). Unlike previous studies that focused mainly on systemic and performance parameters, our work provides a novel investigation into the direct interaction of HA with the GIT mucosa of sheep, using histopathology, immunohistochemistry, and scanning electron microscopy (SEM). Given the dearth of studies examining the action of HA on sheep, the objective of this research is to evaluate the potential beneficial effects of HA on performance and the gastrointestinal tract (GIT) of lambs.

## Materials and methods

### Study site and ethical statement

This study was conducted in accordance with international guidelines for animal welfare and was approved by the Animal Ethics Committee (AEC) of the Pontifícia Universidade Católica do Paraná (PUCPR) under registration number 02061. The study was conducted in the sheep sector of the experimental farm of PUCPR, situated in the municipality of Fazenda Rio Grande, Paraná, Brazil (25.66° S, 49.27° W). The climate is classified as Cfb, which denotes a subtropical highland climate. It is characterised by a mild summer, the absence of a dry season and uniform rainfall distribution. The region experiences severe and frequent frosts (Nitsche et al. 2019).

### Experimental animals

**Fig. 1:**
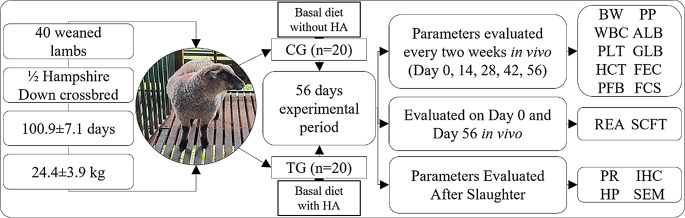
Schematic representation of the experimental design. Lambs were allocated to a Control Group (CG; *n* = 20) receiving a basal diet without a feed supplement, or a Treatment Group (TG; *n* = 20) receiving the basal diet supplemented with humic acid (HA) at 500 mg/kg/day for 56 days. The parameters evaluated are indicated by their abbreviations: ALB, Albumin; BW, Body Weight; CG, Control Group; FEC, Faecal Egg Count; FCS, Fecal Consistency Score; GLB, Globulin; HCT, Haematocrit; HP, Histopathological parameters; IHC, Immunohistochemical parameters; PFB, Plasma Fibrinogen; PLT, Platelet Count; PP, Plasma Protein; PR, Parasite Recovery; REA, Rib Eye Area; SCFT, Subcutaneous Fat Thickness; SEM, Scanning Electron Microscopy; TG, Treatment Group; WBC, White blood cell differential count

The study included 40 weaned Hampshire down crossbred lambs (20 males and 20 females) aged 100.9 ± 7.1 days and an average body weight of 24.4 ± 3.9 kg. The lambs were administered a dose of broad-spectrum anthelmintic monepantel (Zolvix™) at a dosage of 2.5 mg/kg 21 days before the beginning of the experiment. All lambs had no history of metabolic or concurrent ailments and were housed under identical conditions throughout the study period of 56 days. Due to physical space limitations, the 40 lambs were enrolled in two sequential batches. The first batch consisted of 20 lambs, which were randomly allocated into a control group (CG; *n* = 10) and a treatment group (TG; *n* = 10), balanced by weight and sex. Upon completion of the 56-day trial for the first batch, the experiment was repeated with the second batch of the remaining 20 lambs, which were allocated using the same randomization and balancing criteria. Data from both batches were combined for statistical analysis after confirming no significant between-batch effects. The CG was fed a basal diet (Supplementary Table S1) without humic acid (HA) supplementation. The TG received the same basal diet supplemented with a commercial HA product (CLK-WH67; Pharmawerk Weinböhla GmbH, Weinböhla, Germany), which is processed from selected lignites and contains > 65% humic acids. The HA was provided as a powder and mixed with the concentrate daily at a dose of 500 mg/kg body weight/day, which aligns with the minimum oral dosage recommended by the European Agency for the Evaluation of Medicinal Products (EMEA 1999). The overall experimental design is summarized in Fig. 1.

The lambs were maintained in paddocks, with *Lolium multiflorum* Lam. and *Avena sativa* L. pastures during the day throughout the 56-day experimental period to get naturally infected from the first day of experiment on by gastrointestinal parasites. The lambs were housed individually in pens in the evening. In the pens, the lambs received a basal diet of corn silage and concentrate feed, which met the nutritional requirements of sheep (NRC 1985). The concentrate was adjusted weekly, according to lamb weighing data, supplied individually in the amount of 1.0% of the body weight in dry matter (DM) until D35, then the amount supplied was increased to 2.0%. They had access to water *ad libitum*.

### Lamb performance

The body weight (BW) of lambs was measured every week using a YDTech TCS-300 balance (300 kg capacity with 2 g precision). The average daily weight gain (ADWG) was calculated as the difference between the final and initial BW divided by the number of days on the feed.

Rib eye area (REA) and subcutaneous fat thickness (SCFT) were measured in vivo on D0 and D56 with a Sonoscape ultrasound, model Eub-405, with a 12 cm linear transducer length at a frequency of 5 Mhz, using the technique described by Cartaxo et al. (2011) and McManus et al. (2013).

### Haematological and serum biochemical parameters

Four milliliters of blood were collected from each lamb via jugular venipuncture before starting the experiment (D0), on the 14th day (D14), on the 28th day (D28), on the 42nd (D42), and the 56th day (D56). The collected blood was added to plain tubes (including without anticoagulants) for serum biochemical analyses and to other tubes containing EDTA to determine the hematocrit level. Blood samples were placed in a micro-hematocrit centrifuge (Model Spin-1000) for five min at 12,000 rpm. After centrifugation, the capillaries were compared with a standard table to obtain the hematocrit level. Total plasma protein (TPP) concentration was determined using refractometry and plasma fibrinogen (PFB) concentration was measured in g/dL using the heat precipitation method (Bassert 2021). The serum was frozen and transported to the laboratory for further processing. Samples containing serum were processed on Selectra Pro S Biochemistry analyzers, using commercial test kits (Albumin Elitech R and Total Protein Plus Elitech R) to measure total protein (TP) and albumin (ALB). The concentration of globulin (GLB) was calculated as the difference between the total serum protein and albumin. Differential white blood cell (WBC) and platelets count were performed after May-Grünwald/Giemsa-Romanowski staining, following protocols by Dasgupta et al. (2020) and Bassert (2021).

### Fecal consistency score

Fecal consistency was evaluated biweekly using a fecal consistency score (FCS) on a 0-to-4 scale. The procedure involved collecting feces directly from the rectal ampulla of each lamb, followed by visual inspection on a flat surface. Scores were assigned as follows: 0 for normal feces (dry, well-formed pellets), 1 for normal/pasty feces (moist and clumped pellets), 2 for pasty feces (soft, having lost pellet shape but maintaining their form), 3 for diarrheic feces (liquid and unable to hold their form), and 4 for watery diarrhea (completely liquid) (Rosalinski-Moraes et al. 2012).

### Parasitological evaluation

On days 0, 14, 28, 42, and 56 (biweekly), lambs’ feces from the rectal ampulla were stored in plastic bags for fecal egg count (FEC) counting, according to the modified Gordon and Whitlock (1939) method, sensitive to 50 eggs per gram of feces (EPG). Coproculture, according to the method of Roberts and O’Sullivan (1950), was performed with a samples pool from each experimental group to obtain third-stage larvae and identify the parasitic genera according to the morphometry proposed by Wyk and Mayhew (2013). After slaughter, the abomasum was removed and its content processed according to the method of Wood et al. (1995). All parasites of a 10% subaliquot of the total content was counted and identified according to their morphological characteristics (Ueno and Gonçalves 1998).

### Histopathological evaluation and immunohistochemistry

Three pieces (2 × 2 cm) of each tissue from the fundic abomasal region, duodenum (*pars descendens*), jejunum (middle of the length), ileum, caecum (just behind the ileocaecal valve) were collected from the slaughtered lambs of the first batch (CG = 10, TG = 10). This batch was pre-designated for *post-mortem* analyses, and its representativeness was ensured as both sequential batches were balanced for the same initial characteristics (weight, sex, and age). The fragments were immediately stored in 10% formalin for fixation for 24 h. Subsequently, the samples were processed according to the method of Nóbrega et al. (2014). The histological stained slides were scanned using the automated microscope Axio Scan.Z1 at ×400 magnification. To evaluate mucus production in the GIT (abomasum, duodenum, jejunum and ileum), the tissues were stained with periodic acid-Schiff (PAS) technique (Samuelson 2006). The mucus was quantified (%) in ten random areas of 0,17 mm^2^ (in mucosae) using Image-Pro Plus software version 4.5. Eosinophil and mononuclear cells were counted for each tissue sample stained with Giemsa-Romanowski in 5 locations of the 0.1 mm^2^ in each section of mucosa. Abomasal mucosa thickness was measured in 10 regions of tissue (Scott et al. 2017). Count cells and mucosal depth were analysed by computer software Zen 3.9 blue edition.

Similarly, tissues (CG = 10, TG = 10) collected for histology were evaluated by immunohistochemistry using the method of Paula et al. (2021). The samples were incubated with monoclonal mouse anti-human CD4 (Clone 4B12 Dako - code IR649) and monoclonal mouse anti-human CD8 (Clone C8/144B Dako - code IR623) antibodies. The slides were scanned utilizing a similar methodology to that employed for the histological slides. Thereafter, the cells were counted in 10 fields of the 0.1 mm^2^ in each mucosa section, using the Zen 3.9 blue edition software.

### Scanning electron microscopy

A descriptive and exploratory analysis using scanning electron microscopy (SEM) was conducted to investigate the interaction between HA and the gastrointestinal mucosa. The procedure was performed in two stages. First, the pure HA product was directly evaluated to characterize its morphology and serve as a visual reference. Second, from each gastrointestinal segment (abomasum, duodenum, jejunum, ileum, and cecum), two tissue fragments (4 mm² each) were collected from a representative subset of slaughtered lambs (*n* = 2 from CG; *n* = 3 from TG) to search for evidence of adhered HA particles on the mucosal surfaces. The tissues and HA were stored in individual containers containing Karnovsky’s solution for fixation, processed as described by Heuschkel et al. (2019) observed under a JEOL JSM 6010PLUS-LA scanning electron microscope (Microscopy Facility RPT07C PDTIS/Carlos Chagas Institute, FIOCRUZ‐PR) at 20 kV acceleration voltage.

### Statistical analyses

The normality of all variables was evaluated by the Shapiro-Wilk test. For comparisons between two independent groups, the Student’s t-test was applied when assumptions of normality and homoscedasticity were met; otherwise, the Mann–Whitney U test was used. The descriptive statistics are expressed as mean and standard deviation. The repeated measures ANOVA was used to analyse the repeated measures, in which sphericity was analysed by Mauchly’s sphericity test and corrected by Huynh-Feldt or Greenhouse-Geisser correction, according to the epsilon value. Tukey’s multiple comparison test was used to determine which means were different, after applying ANOVA. Statistical analyses were subsequently conducted to assess any potential between-batch effects. FEC data were log-transformed [log10(x + 1)] to normalize the distribution and stabilize variances, but are presented as the original, untransformed means in tables and figures for clarity of interpretation. P-values lower than 0.05 were considered significant. All statistical procedures were conducted through a statistical software program (SPSS, version 25, Armonk, NY: IBM). The SEM analysis was qualitative and descriptive in nature. Therefore, its findings are presented observationally and were not subjected to statistical comparison.

## Results

### Animal performance

Supplemental HA did not affect the growth performance of the lambs. The weight, ADWG, REA and SCFT of the lambs were similar between the groups (Table 1). Throughout the experiment, there was no leftovers of concentrate in any experimental group.

**Table 1:** Influence of humic acid supplementation on growth performance of lambs

Variable	CG	TG	*p* - value
Initial weight (kg)	24.5 ± 4.1	24.3 ± 3.7	0.829
Final weight (kg)	34.0 ± 4.9	32.5 ± 4.5	0.292
ADWG (g/d)	169.7 ± 120.8	146.0 ± 119.5	0.079
REA (cm^2^)	7.0 ± 1.7	7.1 ± 1.3	0.849
SCFT (mm)	2.6 ± 0.8	2.4 ± 0.8	0.483

ADWG = average daily weight gain. REA = rib eye area. SCFT = subcutaneous fat thickness. CG = control group, TG = treated group, supplemented with 500 mg/kg body weight/day of humic acid

### Haematological and serum biochemical parameters

**Fig. 2:**
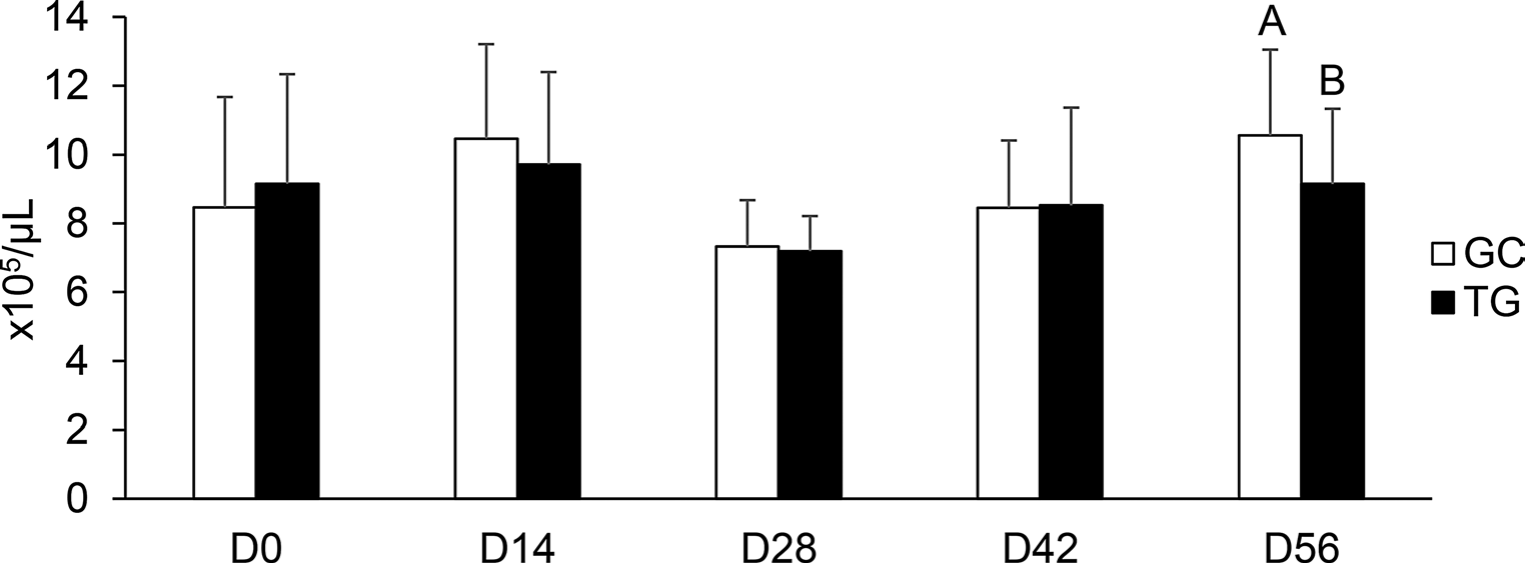
Average number of platelets in whole blood of lambs. CG = control group, TG = treated group, supplemented with 500 mg/kg body weight/day of humic acid. Different letters indicate a significant difference (*p* < 0.05) between groups, as determined by Student’s t-test

In the current study, the WBC, TP, TPP, PFB, ALB, GLB and hematocrit of the lambs were not affected by HA supplementation (Supplementary Table S2). However, platelets decreased (*p* = 0.02) at D56 in TG (Fig. 2).

### Fecal consistency score

**Fig. 3:**
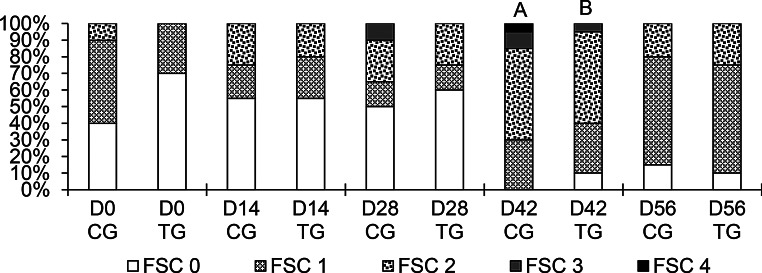
Proportion of the fecal consistency score (FSC) of lambs. CG = control group, TG = treated group, supplemented with 500 mg/kg body weight/day of humic acid. Different letters indicate a significant difference (*p* < 0.05) between groups, as determined by Mann-Whitney U test

The FSC was not altered on the days evaluated, except for D42, The TG demonstrated a higher percentage of better scores (*p* < 0.05) in comparison to the CG (Fig. 3).

### Parasitological evaluation

The genus *Haemonchus* (CG = 96%, TG = 93.8%) was the most prevalent in coproculture, followed by the genus *Trichostrongylus* (CG = 4%, TG = 6.2%). FEC values were similar between CG and TG. The recovery values of larval and adult nematodes showed no significant differences between the groups (Table 2).

**Table 2:** Influence of humic acid supplementation on the number of worms and fecal egg count (FEC) in the Gastrointestinal tract of lambs

Variable	CG	TG	*p* - value
FEC (EPG)	1262.5 ± 2037.5	1007.01 ± 1259.4	0.289
Abomasum			
L4	346.0 ± 256.0	501.1 ± 574.1	0.449
*Haemonchus contortus* female	1288.0 ± 1416.9	884.4 ± 831.6	0.466
*Haemonchus contortus* male	984.0 ± 1169.7	694.4 ± 662.9	0.522
*Trichostrongylus* sp. female	317.0 ± 517.1	172.2 ± 170.1	0.435
*Trichostrongylus* sp. male	293.0 ± 574.4	144.4 ± 125.6	0.459
Total number of worms	3228.0 ± 3411.1	2396.7 ± 1995.5	0.532
Small intestine			
*Trichostrongylus colubriformis* female	223.0 ± 273.2	215.6 ± 221.8	0.949
*Trichostrongylus colubriformis* male	92.0 ± 120.8	98.9 ± 99.0	0.894
Total number of worms	315.0 ± 392.8	314.4 ± 317.7	0.997

EPG = eggs per gram of feces. L4 = fourth stage larvae. CG = control group, TG = treated group, supplemented with 500 mg/kg body weight/day of humic acid

### Histopathological evaluation and immunohistochemistry

No significant differences in the abomasal mucosa thickness and the percentage of mucus produced in the GIT of lambs were observed between the groups (Supplementary Table S3).

**Fig. 4:**
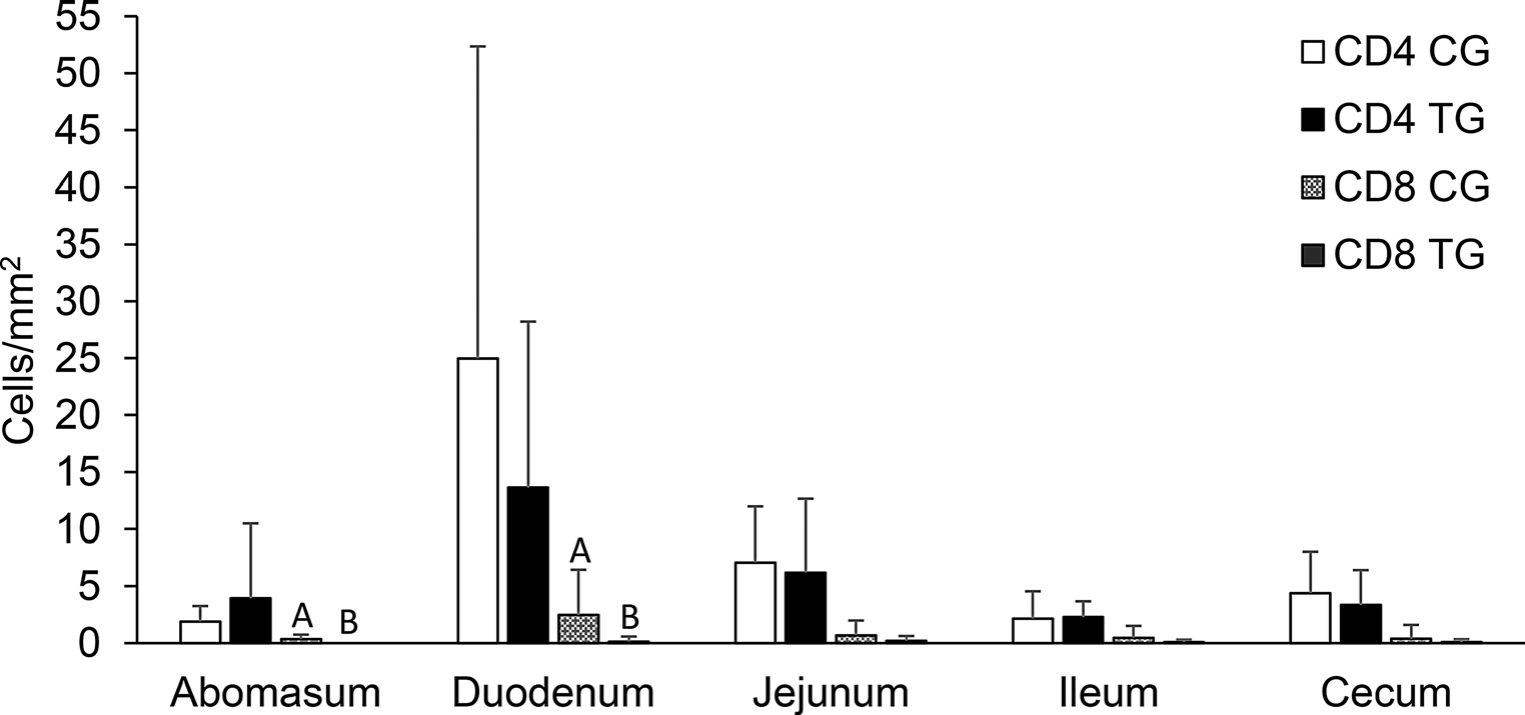
Average number of CD4 and CD8 cells in the gastrointestinal mucosa of lambs. CG = control group, TG = treated group, supplemented with 500 mg/kg body weight/day of humic acid. Different letters indicate a significant difference (*p* < 0.05) between groups, as determined by Student’s t-test

**Fig. 5:**
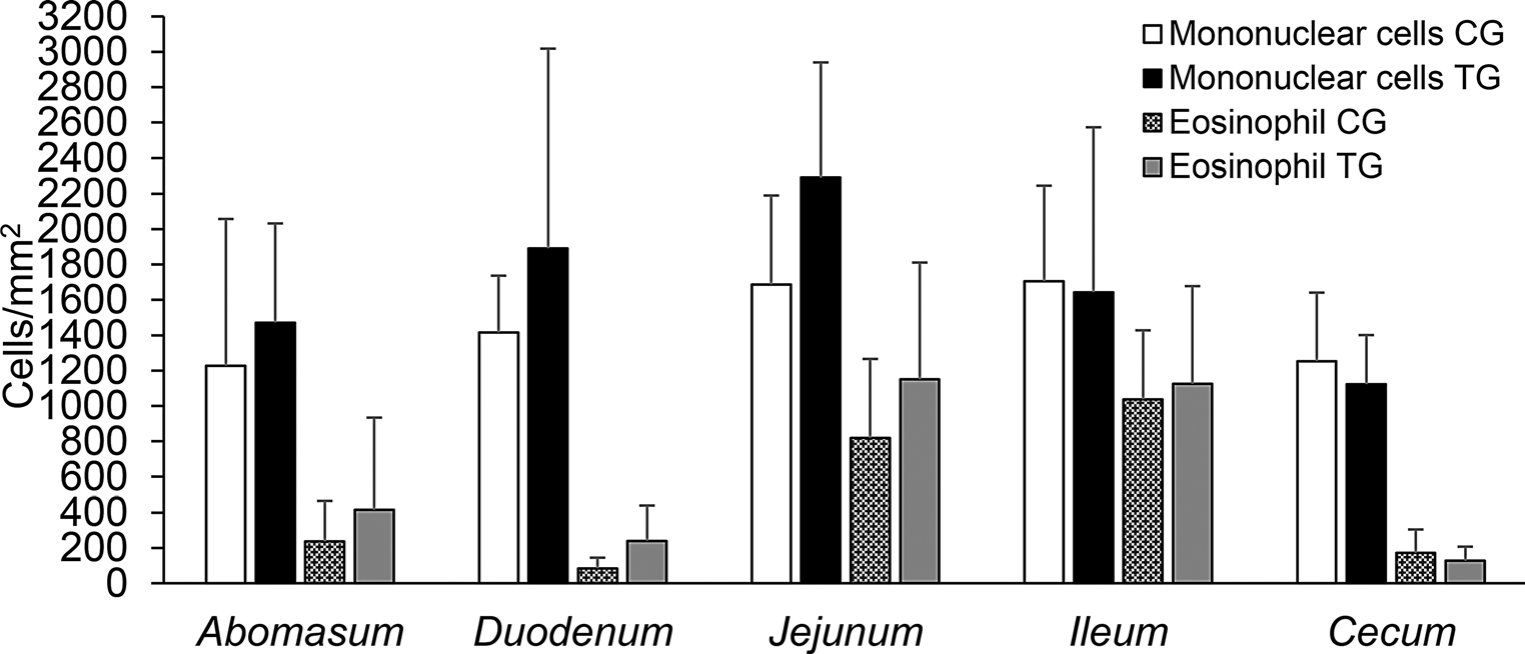
Average number of mononuclear cells and eosinophils in the gastrointestinal mucosa of lambs. CG = control group, TG = treated group, supplemented with 500 mg/kg body weight/day of humic acid

There was no difference in the CD4 cells count in the GIT mucosa between the groups. Likewise, HA supplementation did not change the values of CD8 cells in the jejunum, ileum and cecum, however in the mucosa of the abomasum and duodenum in the TG, CD8 cells were decreased (*p* < 0.05) compared to the CG (Fig. 4). Eosinophils and mononuclear cells in the GIT mucosa were not affected by the addition of HA to the lamb diet (Fig. 5).

### Scanning electron microscopy

**Fig. 6:**
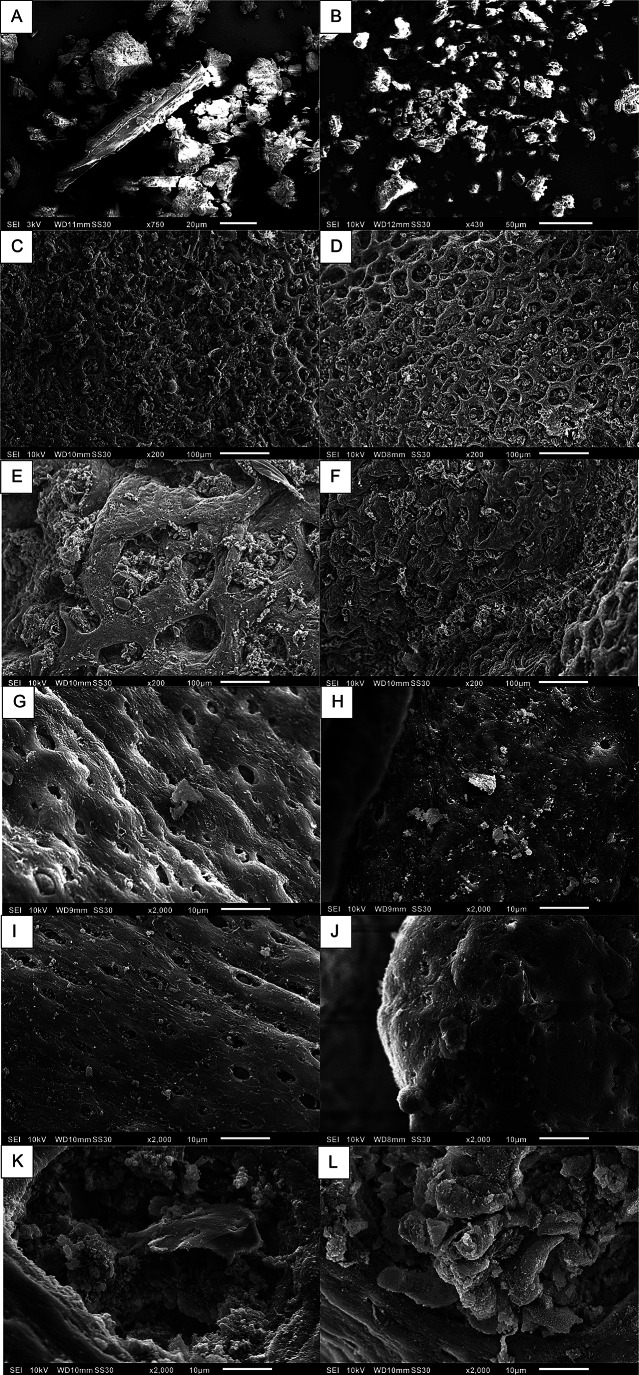
Scanning electron microscopy of the pure humic acid (HA) supplement and representative mucosal surfaces from the gastrointestinal tract of lambs from the control (CG) and treated (TG) groups. The pure HA supplement at low (**A**) and high (**B**) magnification, showing its heterogeneous morphology with particles of different shapes and sizes. Abomasal mucosa from CG (**C**) and TG (**D**), exhibiting similar characteristic gastric pit openings. Duodenal mucosa from CG (**E**) and TG (**F**), showing a complex surface covered by a bacterial biofilm. High-magnification view of the jejunal surface from CG (**G**) and TG (**H**). High-magnification view of the ileal surface from CG (**I**) and TG (**J**). Detailed views of the cecal mucosa from CG (**K**) and TG (**L**). Magnifications and scale bars: (**A**) 430x, 50 μm; (**B**) 750x, 20 μm; (**C**-**F**) 200x, 100 μm; (**G**-**L**) 2000x, 10 μm

It was not possible to find structures that could be definitively identified as humic acid particles on the GIT surface. The histological analysis did not reveal any HA deposits (Supplementary Figure S1), and similarly, SEM did not confirm the presence of adhered HA particles. Instead, the SEM analysis showed that the mucosal surface across all evaluated segments was complex, often covered by a diverse bacterial biofilm and adhered particulate matter, making it difficult to distinguish the supplemented HA (Fig. 6).

## Discussion

Several studies have indicated that HA supplementation can have beneficial effects on the health and performance of a range of domestic animals. However, it should be noted that the response may vary depending on the dosage regimen and animal species (Islam et al. 2005; Cusack 2008; Zralý and Písaříková 2010; Ozturk et al. 2012; Nagaraju et al. 2014; Wang et al. 2020a, 2022b).

An important question is whether HA are absorbed by the mucosa or persist within the gastrointestinal wall to perform their functions. Since HA have a high molar mass (5,000–100,000 Da), they remain almost completely in the GIT after oral administration (Islam et al. 2005). However, according to Stein (1994), the penetration of HA into epithelial tissue is desirable for the better manifestation of its biological activity. Our study was the first to attempt to identify HA in the GIT mucosa of ruminants using SEM and histopathology. However, histological and SEM analyses did not identify specific particles of this compound within the GIT mucosa reinforcing that the high molecular weight hinders absorption. SEM presented a particular challenge because HA exhibited a variety of shapes and sizes, making it difficult to differentiate from other structures (Fig. 6). The absence of HA in the mucosa stands in contrast to the observations reported by Buesing et al. (2011) in pigs, where HA particles were identified in histological samples of the tissue layers of the gastrointestinal tract (epithelial cells, lamina propria, submucosa, and muscularis) and bladder (urothelial cells, lamina propria, submucosa, and muscularis). In contrast, our study did not identify such particles in sheep. The hypothesis proposed by Buesing et al. (2011) suggests that HA absorption may have occurred through the lymphatic and circulatory systems to the urinary tract. The discrepancy between the present study and that of Buesing et al. (2011) may be attributed to the distinct characteristics of the gastrointestinal tracts of monogastrics and ruminants. These findings highlight a possible fundamental difference in HA absorption in the GIT between pigs and ruminants, suggesting that in ruminants, HA may remain in the rumen benefiting bacteria, and, consequently, an insufficient amount of HA continues to the intestine (Terry et al. 2018; Kholif et al. 2021) to eventually be absorbed.

The immune system is extremely important in the fight against infection in the GIT of sheep (Lacroux et al. 2006; Hassan et al. 2022). The protection against infection by gastrointestinal nematodes is mediated by type 2 immune responses, which involve cytokine induction and antibody production, as well as the mobilization of innate and adaptive immune cells (Shakya et al. 2009; Yang et al. 2015). The present study did not reveal any alterations in systemic immunological parameters between the CG and TG. The results of this experiment were within the reference values for sheep. One possible explanation for this is that the parasitic infection was not sufficiently severe to alter the WBC values (Bastos et al. 2016). Some studies have reported a reduction in the FEC in goats fed with HA, with the hypothesis that this effect on parasitosis may be attributed to host immunostimulation (El-Zaiat et al. 2018; Ikyume et al. 2022). However, these studies did not specify which parasite species were affected. In our study, it has been observed that HA did not exhibit activity against *Haemonchus contortus*, which constituted the largest population of parasites identified, neither through a direct effect on the parasite nor via immune modulation. These findings suggest that *H. contortus* may have been abundant less than other nematode species or even absent in the samples analysed in previous studies (El-Zaiat et al. 2018; Ikyume et al. 2022; Sallam et al. 2023). In the present study, no effect of HA supplementation was observed on parasitological and immunological parameters, except for a reduction in CD8 cells in the mucosa of the abomasum and duodenum. The precise mechanism of HA interaction with the gastrointestinal mucosa remains unclear. A reduction in CD8 cells is contrary to the findings of Vucskits et al. (2010), who observed an increase in Peyer’s plaques and general immune function in rats. However, direct comparisons should be made with caution due to the known physiological differences between monogastric and ruminant species. It may be postulated that this is an effect of immune modulation (anti-inflammatory effect) rather than immune stimulation (Van Rensburg 2015). Also, it is hypothesised that HA could reduce the levels of pro-inflammatory cytokines as TNF-α and IL-6, which are normally increased in endoparasitic infections (Schafer et al. 2015; Wang et al. 2020a). This potential effect of HA on cytokines could influence the quantity of CD8 cells in the GIT mucosa (Cash et al. 2010).

Similar results for performance parameters were observed between CG and TG. These results are in accordance with Budak et al. (2022); Terry et al. (2018); Wang et al. (2020b), who used HA in the ruminant diet and found no difference in weight gain. However, Burezq and Khalil (2022) observed in their study that lambs supplemented with 2.5 g of HA/animal/day gained more weight compared to the group that did not receive any type of additive. The HA dose of 500 mg/kg body weight/day used in our study aligns with the minimum oral dosage recommended by the EMEA (1999). Moreover, our protocol achieves greater precision by individualizing the dose per animal, compared to studies that administer fixed amounts regardless of body weight (El-Zaiat et al. 2018; Terry et al. 2018; Ataollahi et al. 2024) or those based solely on feed proportion (Wang et al. 2020b). Differences between experiments may be explained by the fact that the chemical composition and concentration of HA varied according to geographical origin and process of obtainment (Pospíšilová et al. 2015; Melo et al. 2016). Another potential explanation is the variation in the microbiological environment of the farms, as proposed by Vucskits et al. (2010). In this context, a more challenging bacterial environment may benefit from the immune-stimulating effects of HA (Jooné and Van Rensburg 2004; Wang et al. 2008; Cetin et al. 2011; Mudroňová et al. 2020).

The values of biochemical parameters were similar in this study between groups. However, it was observed that platelet values in the HA-supplemented group were reduced at D56, although remaining well within the physiological reference range for the species, which may indicate a potential to modulate the immune response and reduce inflammation. One hypothesis for this finding is that the supplemented humic acids induced this platelet reduction through a mechanism similar to that described by Lan et al. (2022). According to the authors, the inhibition of Protein Disulfide Isomerase (PDI), an enzyme present in exosomes released by endothelial cells, leads to a lower activation of the glycoprotein IIb-IIIa complex on the platelet surface. Since the activation of GPIIb-IIIa is the crucial step for platelet aggregation, and this aggregation is, in turn, the main trigger for degranulation and the release of pro-inflammatory mediators, interference in this pathway represents a potent anti-inflammatory mechanism. Therefore, the action of humic acids on this axis may justify both the observed modulation in platelet values and their potential to attenuate the inflammatory response. The observation of this effect only at the final time point suggests a time-dependent mechanism. This could be due to a cumulative effect of the supplement or a requirement for a prolonged period of administration before a response is elicited. A longer experimental period would be valuable to determine if this platelet-modulating effect persists or strengthens over time.

On day 42, fecal consistency exhibited a range from pasty to watery, a change attributed to the increased lamb concentrate introduced on day 35. This resulted in alterations to gastrointestinal transit dynamics, leading to the production of watery stools (Chen et al. 2023). Nevertheless, despite this dietary modification, lambs administered with HA exhibited a significantly lower fecal score compared to the CG. The absence of significant differences at other evaluation times is likely because the animals did not face a similar diarrheic challenge, thus the protective effect of HA became evident only when gastrointestinal transit was altered by the dietary change, doubling the concentrate, from day 35 onwards. This suggests that the benefit of HA on fecal consistency is most apparent under conditions of moderate gastrointestinal stress. Our findings are in line with those of Wang et al. (2022b), who reported that calves supplemented with HA had a lower incidence of diarrhea. One proposed mechanism for this anti-diarrheal effect is the ability of HA to establish a protective barrier on the epithelial mucosa, facilitated by their colloidal properties (He et al. 2023; Xu et al. 2023). However, since our direct microscopy analyses failed to identify adhered HA particles on the mucosal surface, the physical barrier hypothesis is not supported by our findings. Therefore, it is more plausible that the observed reduction in fecal scores resulted from the other recognized properties of HA, such as its local anti-inflammatory and immunomodulatory effects (Van Rensburg et al. 2000; Jooné and Van Rensburg 2004).

The presence of abomasal parasites has been noted to induce morphological changes in the host mucosa, characterized by cell hyperplasia, superficial epithelial damage, and depletion of acid-producing parietal cells (Rinaldi et al. 2011; Simpson et al. 2016; Scott et al. 2017). While the mucus produced in the GIT serves as the primary defence against parasitic infections, its specific role in response to abomasal parasites remains inadequately defined (Rinaldi et al. 2011; Hasnain et al. 2013). In our study, no discernible differences were observed in the size of the abomasal tissue layer or mucus production between experimental groups. However, prior investigations suggest that augmented production of mucus (glycoprotein) within the GIT may correlate with the host’s ability to expel nematodes from this environment (Li et al. 2009; Hoang et al. 2010; Menzies et al. 2010; Takeda et al. 2010). The administration of HA did not result in any discernible alterations in mucus production, histological measurements, or the parasitic burden within the GIT, thereby reinforcing the notion that HA exerts no discernible influence upon the GIT tissue.

In conclusion, HA reduced the number of platelets and CD8 values in the abomasum and duodenum, indicating possible modulation in the immune response and reduction of local inflammation. In addition, fecal consistency improved, which also is a result of less inflammation. HA were not found in the mucosa of the gastrointestinal system of lambs. However, under the conditions of this study, HA supplementation did not significantly affect growth performance, parasitological or most of the haematological and biochemical parameters.

## Supplementary Information

Below is the link to the electronic supplementary material.

Supplementary Material 1

## Data Availability

The data sets generated and/or analyzed during the current study are available through the corresponding author upon reasonable request.
