# Supplementary material for: Effect of humic acid supplementation on lamb gastrointestinal health and performance
Source: Trop Anim Health Prod. 2026 Feb 12;58(2):102. doi: 10.1007/s11250-026-04882-5 (PMC12901293; doi:10.1007/s11250-026-04882-5)
Supplement: Supplementary file 1 — Supplementary Material 1 [file 11250_2026_4882_MOESM1_ESM.docx]

| Supplementary Table S1. Composition of the basal diet of lambs. | |
| --- | --- |
| Item | Quantity |
| Corn silage |  |
| Dry matter (%) | 39.00 |
| Crude protein (%) | 6.20 |
| Neutral detergent fiber (%) | 40.40 |
| Starch (%) | 38.80 |
| Ether extract (%) | 3.20 |
| Mineral matter (%) | 2.80 |
| Non-fibrous carbohydrate (%) | 48.50 |
| Total digestible nutrients (%) | 69.00 |
| Concentrate (Coasul^®^) |  |
| Crude Protein (%) | 14.0 |
| Ether Extract (%) | 2.5 |
| Mineral Matter (%) | 10.0 |
| Crude Fiber (%) | 14.0 |
| Acid Detergent Fiber (%) | 14.0 |

| Supplementary Table S2. Influence of humic acid supplementation on hematological and serum biochemical parameters of lambs. | | | | | | |
| --- | --- | --- | --- | --- | --- | --- |
| Variable | Groups | D0 | D14 | D28 | D42 | D56 |
| Hematocrit (%) | CG | 31.6 ± 3.6 | 32.0 ± 2.2 | 29.5 ± 3.0 | 25.8 ± 3.0 | 26.5 ± 2.4 |
|  | TG | 31.9 ± 3.5 | 32.0 ± 3.0 | 29.2 ± 3.3 | 27.0 ± 3.4 | 27.0 ± 2.6 |
|  |  |  |  |  |  |  |
| Total plasma protein (g/dL) | CG | 6.7 ± 0.3 | 6.8 ± 0.4 | 6.9 ± 0.4 | 6.4 ± 0.6 | 6.5 ± 0.6 |
|  | TG | 6.7 ± 0.4 | 6.8 ± 0.4 | 6.8 ± 0.6 | 6.6 ± 0.7 | 6.6 ± 0.6 |
|  |  |  |  |  |  |  |
| Total protein (g/dL) | CG | 5.8 ± 0.4 | 5.9 ± 1.1 | 5.3 ± 0.9 | 5.4 ± 1.1 | 6.0 ± 0.7 |
|  | TG | 5.6 ± 0.5 | 5.9 ± 1.1 | 5.2 ± 0.8 | 5.4 ± 0.9 | 5.8 ± 0.4 |
|  |  |  |  |  |  |  |
| Albumin (g/dL) | CG | 2.4 ± 0.1 | 2.5 ± 0.3 | 2.6 ± 0.4 | 2.4 ± 0.3 | 2.3 ± 0.2 |
|  | TG | 2.3 ± 0.2 | 2.6 ± 0.3 | 2.6 ± 0.4 | 2.3 ± 0.2 | 2.3 ± 0.2 |
|  |  |  |  |  |  |  |
| Globulin (g/dL) | CG | 3.3 ± 0.4 | 3.4 ± 1.2 | 2.6 ± 1.1 | 2.9 ± 1.2 | 3.7 ± 0.6 |
|  | TG | 3.2 ± 0.4 | 3.3 ± 1.3 | 2.6 ± 1.1 | 3.1 ± 0.9 | 3.5 ± 0.4 |
|  |  |  |  |  |  |  |
| Plasma fibrinogen (g/dL) | CG | 0.2 ± 0.2 | 0.2 ± 0.2 | 0.2 ± 0.1 | 0.2 ± 0.2 | 0.2 ± 0.1 |
|  | TG | 0.2 ± 0.1 | 0.3 ± 0.2 | 0.2 ± 0.2 | 0.2 ± 0.2 | 0.3 ± 0.1 |
|  |  |  |  |  |  |  |
| Neutrophils (%) | CG | 45.4 ± 11.4 | 40.5 ± 13.0 | 36.6 ± 13.8 | 39.0 ± 15.0 | 42.3 ± 12.7 |
|  | TG | 42.5 ± 12.0 | 40.3 ± 9.3 | 34.8 ± 9.5 | 39.1 ± 9.5 | 41.7 ± 15.4 |
|  |  |  |  |  |  |  |
| Lymphocytes (%) | CG | 52.8 ± 11.8 | 52.4 ± 14.3 | 57.9 ± 14.9 | 57.6 ± 18.3 | 55.5 ± 13.4 |
|  | TG | 56.3 ± 12.0 | 54.6 ± 11.5 | 61.0 ± 9.54 | 57.2 ± 11.9 | 57.2 ± 15.6 |
|  |  |  |  |  |  |  |
| Monocytes (%) | CG | 1.0 ± 1.6 | 0.5 ± 0.9 | 0.2 ± 0.5 | 0.2 ± 0.5 | 0.4 ± 0.8 |
|  | TG | 0.7 ± 0.7 | 0.3 ± 0.7 | 0.2 ± 0.4 | 0.5 ± 0.7 | 0.1 ± 0.4 |
|  |  |  |  |  |  |  |
| Eosinophils (%) | CG | 0.8 ± 1.1 | 6.5 ± 6.5 | 5.3 ± 5.4 | 3.1 ± 5.2 | 1.7 ± 1.9 |
|  | TG | 0.5 ± 0.7 | 4.6 ± 3.8 | 3.9 ± 3.7 | 3.1 ± 4.3 | 0.9 ± 1.4 |
| CG = control group. TG = treated group. supplemented with 500 mg/kg body weight/day of humic acid. | | | | | | |

| Supplementary Table S3. Influence of humic acid supplementation on histological analysis of the gastrointestinal tract of lambs. | | | |
| --- | --- | --- | --- |
| Variable | CG | TG | p - value |
| Abomasal mucosa thickness (µm) | 767.7 ± 101.8 | 685.1 ± 138.4 | 0.146 |
| Abomasum mucus (%) | 15.1 ± 7.8 | 14.8 ± 5.9 | 0.207 |
| Duodenum mucus (%) | 4.9 ± 2.8 | 7.2 ± 5.1 | 0.223 |
| Jejunum mucus (%) | 3.6 ± 1.7 | 6.9 ± 11.0 | 0.360 |
| Ilium mucus (%) | 5.5 ± 3.7 | 8.9 ± 10.4 | 0.342 |
| CG = control group. TG = treated group. supplemented with 500 mg/kg body weight/day of humic acid. | | | |
